# Evaluation of Notch1 and CD10 Expressions in Colorectal Carcinoma and Their Relationship with Prognosis 

**DOI:** 10.30699/ijp.2024.2029781.3304

**Published:** 2025-01-10

**Authors:** Noha Elkady, Reham Ahmed abdelaziz, Rania Abdallah

**Affiliations:** 1 *Pathology Department, Faculty of Medicine, Menoufia University, Shibin Elkom, Menoufia, Egypt *; 2 *Clinical Oncology and Nuclear medicine Department Faculty of Medicine, Menoufia University, Shibin Elkom, Menoufia, Egypt*

**Keywords:** Colorectal carcinoma, Notch1, CD10, progression

## Abstract

**Background & Objective:**

Even with improvements of colorectal cancer (CRC) treatment strategies, this cancer still has an unfavorable outcome. The primary cause of CRC development and recurrence is chemoresistance. CD10 and Notch1 are among cancer stem cell regulators, and they have roles in cancer progression and chemoresistance. This research aims to evaluate the expression of Notch1 and CD10 in CRC and their relationship with different clinicopathological parameters using immunohistochemistry.

**Methods:**

This retrospective study included 100 cases of colorectal carcinoma that were immunohistochemically stained using Notch1 and CD10 antibodies. Expression of Notch1 and CD10 was evaluated and compared with different clinicopathological parameters.

**Results:**

Notch1 expression was detected in the tumor and stromal cells in 92% of the cases, while CD10 expression was seen in 31% of tumor cells 79% of stromal cells of the included cases. Their expressions in tumor cells were significantly associated with higher grade (*P*=0.029 and 0.001), deeper invasion (*P*=0.01 and 0.002), advanced stage (*P*=0.012 and 0.001), and distant metastasis (*P*=0.001 and 0.02). Notch1 expression was positively correlated with CD10 expression (*P*=0.018). Both Notch1 expression and high CD10 expression in the stromal cells were associated with short overall survival (*P*=0.003 and 0.01).

**Conclusion:**

CD10 and Notch1 may have roles in colorectal carcinoma progression via induction of tumor invasion, metastasis and impairment of tumor response to therapy. CD10 and Notch1 could be used as biomarkers for aggressive CRC and may be considered for future target therapy.

## Introduction

Colorectal cancer (CRC) is the third most commonly diagnosed cancer in the United States and the second most common cause of cancer-related death worldwide. Its global incidence is on the rise, particularly in individuals under age 55, which may be linked to increased exposure to environmental factors as well as changes in lifestyle and diet (1). In Egypt, the incidence of CRC is approximately 14%, with the peak age between the fifth and seventh decades; notably, 25% of patients are under 40 years old (2).

Surgery is the primary treatment modality; however, chemoradiotherapy is often used either before surgery as neoadjuvant therapy or after surgery as adjuvant therapy to decrease the risk of recurrence and metastasis in advanced CRC. Chemotherapy typically involves different groups of cytotoxic drugs targeting rapidly dividing cells and is often used in combination (3).

Although overall survival of CRC patients has improved with advances in treatment and the introduction of new therapeutic options, prognosis remains poor. Tumor stage and the presence of metastasis are among the most important prognostic factors for CRC (4).

Chemoresistance is a common challenge in cancer, increasing the likelihood of tumor recurrence. It may be attributed to multiple factors, including tumor biology, genetic profiles, and the presence of cancer stem cells (CSCs) (3). CSCs are clusters of tumor cells that resist therapy and raise the risk of recurrence and metastasis (5). They are regulated by several molecules, with Notch1 and CD10 among the main regulators. Investigation on Notch1 and CD10 expression in tumor may help predicting drug resistance (6).

Ultimately, understanding tumor biology is crucial for identifying biomarkers that can predict therapy response. This will thereby aid in the selection of patients who will benefit from neoadjuvant treatment or in the development of targeted therapies.

This study aims to assess the expression of Notch1 and CD10 in CRC through immunohistochemistry (IHC) and to correlate their expression with various clinicopathological features in order to determine their prognostic impact.

## Material and Methods

### Patients and Samples

In this retrospective study, all cases diagnosed as colorectal carcinoma from January 2018 to December 2019 were collected from the pathology department archive, Faculty of Medicine Menoufia University after obtaining ethical approval. The cases with available paraffin blocks (100 cases) were included in this retrospective study. The clinical and follow-up data were retrieved from patients' records. The study was conducted in accordance with the Declaration of Helsinki in 1975 and modified in 2000. 

### Histopathological Evaluation

Hematoxylin and eosin (H&E) stained slides were microscopically examined to confirm the diagnosis and evaluate different histopathological findings, such as tumor grade (a two-tier grading system was used; low and high), depth of tumor invasion, tumor deposit in lymph node, pathological staging (Tumor stages I and II were considered early stages while Stage III and IV were considered advanced stages), and vascular invasion. 

### Tissue Microarrays

After the collection of paraffin blocks, tissue microarrays (TMA) were constructed in which three cores, each 2mm in diameter, were taken from each block by array needle (Beecher Instruments, Silver Spring, USA). The obtained tissue cores were arranged into the recipient blocks, followed by drawing a map indicating the origin of each core.

### Immunohistochemical Staining

From each block, two 4-µm-thick sections were cut then they were placed on positively charged slides, followed by immunohistochemical staining using a streptavidin-biotin amplified system and diaminobenzidine (DAB) chromogen. The process started with deparaffinization of the sections, followed by rehydration and antigen retrieval by boiling the slides in citrate buffer and then cooling them at room temperature. Blocking endogenous peroxidase was done by adding hydrogen peroxide (3%). The 1ry antibodies were added to the sections and incubated overnight, followed by adding the detection kit (Envision, FLEX, code 8002, Dako) and DAB. The primary antibodies were Notch1 antibody (rabbit polyclonal antibody, A18282, concentrated, ABclonal, USA) and CD10 antibody (mouse monoclonal antibody, IR648 RTU FLEX, Dako, Aligens, USA). 

The expression of Notch1 and CD10 was evaluated in tumor and stromal cells. Positive expression was considered when any number of cells showed brown staining. Then, their expression was semi-quantitatively evaluated using H-score. The intensity of staining was described as follows: 0 negative (no staining), 1 mild (faint brown), 2 moderate (pale brown), and 3 strong (dark brown). The percentage of positively stained cells was also determined (0-100%), and then the H-score was calculated by multiplying the above two values. The expression of Notch1 and CD10 was divided into low and high relative to the mean value of the H score. (7)

### Statistical Analysis

Collected data were arranged in some tables, followed by statistical analysis using SPSS 22 (SPSS Inc., Chicago, IL., USA). The Chi-square test (χ^2^), Fisher exact test (F), and Spearman correlation test (r) were used. Overall survival (OS) analysis was done using the Kaplan-Meier method, and a log-rank test was used to compare the differences. P value < 0.05 was considered statistically significant, and a P-value less than 0.001 was considered highly significant.

## Results

### The Clinicopathological Data of the Included Cases

This retrospective study included 100 patients who were diagnosed as colorectal carcinoma (CRC); their ages ranged between 21 and 80, with a mean age of 51.9. CRC included males (58%) more than females. The tumor was found to infiltrate the serosa or extend beyond and present at an advanced stage in more than half of the cases. Metastasis was present in 18% of the cases. (Table 1)

### Notch1 and CD10 Expression in the Studied Cases

**Fig. 1:**
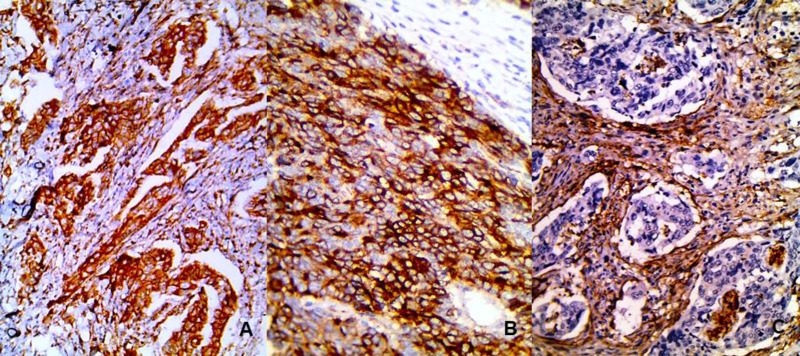
Notch1 and CD10 expressions in CRC. A. Notch1 expression in tumor and stromal cells (200X). B. CD10 expression in tumor cells (400X). C. CD10 expression in stromal cells (200X).

Notch1 expression was detected in tumor and stromal cells of 92% of cases. Its expression appeared as brown staining mostly in the cytoplasm in addition to cell membrane (Figure 1). High Notch1 expression was detected in half of the cases.

### Relationship Between Notch1 Expression and Different Clinicopathological Parameters

Significant associations were observed between high Notch1 expression and high-grade tumors (*P*=0.029), deeper tumor invasion (*P*=0.01), lymph node involvement (*P*=0.011), and advanced tumor stage (*P*=0.012). Moreover, high expression was significantly associated with vascular invasion (*P*=0.014), distant metastasis (*P*=0.001) and partial response to therapy (*P*=0.003) (Table 2 ).

### Relationship Between CD10 Expression and Different Clinicopathological Parameters

CD10 positive expression in tumor cells and high expression in stromal cells were significantly associated with high-grade tumors (*P*=0.001 and 0.024), deeper tumor invasion (*P*=0.002 and 0.007), lymph node metastasis (*P*=0.001 and 0.004) and advanced tumor stage (*P*=0.001 and 0.002), vascular invasion (*P*=0.035 and 0.03) and metastasis (*P*=0.02 and 0.023). In addition, high CD10 expression in stromal cells was also associated with males (*P*=0.002), with larger tumor size (*P*= 0.04), and partial tumor response to therapy (*P*=0.004) (Table 3).

### Relationship Between Notch1 and CD10 Expression

The Spearman test showed a significant positive correlation between the H score of Notch1 and CD10 expressions (p=0.018). 

### Survival Analysis

**Fig. 2:**
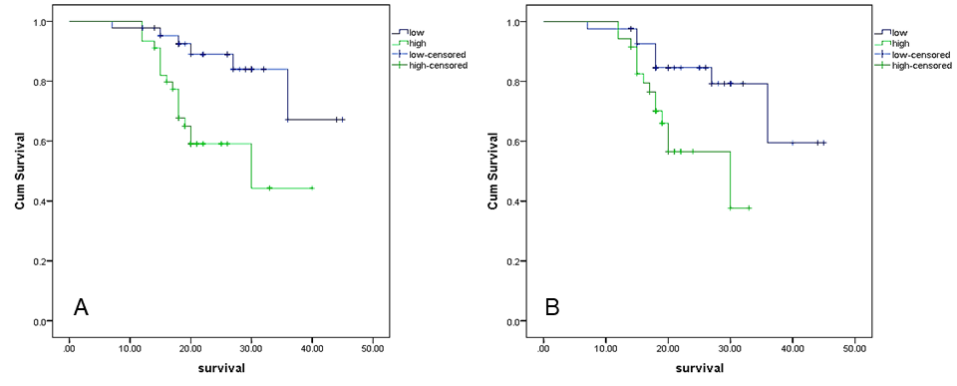
Kaplan Meyer survival analysis showed decreased patients’ survival in association with high Notch1 expression (A), and high CD10 expression in tumor stroma (B).

Overall survival analysis using the Kaplan-Meyer method and log-rank test revealed that high Notch1 and CD10 expression in stromal cells were associated with short overall patients’ survival (P=0.003 and 0.01) (Figure 2). In contrast, none of the variables was found to be independent predictors of survival after analysis using the Cox proportional hazards regression model.

## Discussion

Mortality rate of CRC remains high, potentially due to advanced tumor stage at diagnosis, aggressive tumor biology, or therapy resistance (4, 8). This situation has led to increased investigation into the molecular changes underlying CRC to identify potential targets for therapy. This study aimed to evaluate the expression of Notch1 and CD10, both of which have been implicated in tumor progression and therapy resistance.

In this study, a significant proportion of CRC cases were diagnosed in individuals under the age of 55, which aligned with observations by Vuik et al. (2019). They attributed the rising incidence of CRC in young adults in Europe to changing dietary habits (8).

The study findings also indicated that most cases involved tumor infiltration to the serosa/adventitia or beyond, often accompanied by lymph node metastasis, resulting in an advanced stage at presentation. Similar results were reported in a U.S. study showing an increased incidence of advanced-stage CRC at diagnosis, particularly in younger patients, potentially reflecting aggressive tumor biology or limited screening. Delayed symptom onset is also a factor, as many CRCs are asymptomatic until they have progressed to an advanced stage (9).

Notch1 expression was observed in both tumor and stromal cells in most samples, corroborating reports in the literature documenting Notch1 expression in various cancers and its role in tumorigenesis (10). In this study, high Notch1 expression was significantly associated with high-grade tumors, a finding also noted in prostate cancer (11) and hepatocellular carcinoma (12). Notch1 may drive carcinogenesis by promoting cell proliferation, dysregulating apoptosis, and facilitating angiogenesis (10).

Additionally, high Notch1 expression showed significant associations with deeper tumor invasion, lymph node metastasis, advanced tumor stage, metastasis, and shorter overall survival—corroborating the work of other researchers (11, 12). Notch1 influences invasion and metastasis by promoting epithelial-mesenchymal transition (EMT) through NFκB, TGFβ, Twist, and Slug, conferring migratory ability to epithelial cells. It also activates matrix metalloproteinases (MMPs), which degrade extracellular matrix and stroma, thereby facilitating tumor spread (13, 14).

Among the studied cases, 79 showed CD10 expression in the tumor stroma. High CD10 expression was significantly associated with tumor progression and metastasis—consistent with findings in prostate (15), breast (16), and gastric cancers (17). As one of the metallopeptidases, CD10 may contribute to carcinogenesis by releasing bioactive substances that stimulate tumor cell proliferation and angiogenesis. It promotes invasion and metastasis through matrix degradation and modulates EMT via interaction with the SNAIL gene. CD10 may also alter the immune response against tumor cells (18) and reduce cell adhesion, facilitating invasion (19).

Moreover, high Notch1 and CD10 expression were significantly linked to reduced response to chemotherapy. Other studies similarly identified an association between CD10/Notch1 expression and therapy resistance (20, 21). Notch1 mediates chemoresistance by regulating stem cell differentiation (22), inducing EMT, elevating multidrug-resistance protein 1 (ABCC1/MRP1) expression (20), and enabling continuous DNA repair (21, 23). CD10 expression can induce drug resistance by promoting G0/G1 arrest, increasing transporter pumps, enhancing DNA repair, and inhibiting apoptosis (24), while also regulating stemness through OCT3/4 expression and β1-integrins (25, 26).

Finally, this study suggests a possible cross-talk between Notch1 and CD10 expression that may drive tumor aggressiveness and treatment resistance. Another study also found a significant correlation between Notch1 and CD10 expression (27). CD10 expression is thought to occur via both Notch1-dependent and -independent pathways. Notch1 activates CD10, and both regulate tumor stemness (25). In turn, CD10’s proteolytic activity cleaves the Notch1 receptor, shedding its extracellular domain and further activating Notch1 (28). Based on these findings, Notch1 and CD10 could be potential therapeutic targets in CRC, used in combination with chemotherapy to limit tumor progression and enhance therapy response (29, 30). However, future molecular or in vitro studies are needed to confirm their roles and assess their efficacy as targeted therapies in CRC.

The main limitation of this study was the lack of funding and limited access to advanced research techniques and high-quality equipment, constraining detailed investigations. Nevertheless, larger-scale research using more sophisticated methodologies is recommended to clarify the mechanisms behind Notch1 and CD10 expression in tumor progression and resistance, and to validate their potential as targeted therapies in CRC.

**Table 1:** Clinicopathological data of the studied cases

Clinicopathological parameters	Number (%)
Sex	
Male	58(58%)
Female	42 (42%)
Age	
<55	47 (47%)
>55	53 (53%)
Size	
<6	56 (56%)
>6	44 (44%)
Grade	
Low	66 (66%)
High	34 (34%)
Tumor extent	
T1	2 (2%)
T2	22 (22%)
T3	55 (55%)
T4	21 (21%)
Lymph node	
Negative	45 (45%)
Positive	55 (55%)
Pathological Stage	
Early	48 (48%)
Advanced	52 (52%)
Vascular invasion	
Negative	70 (70%)
Positive	30 (30%)
Metastasis	
Negative	82 (82%)
Positive	18 (18%)
Response to therapy	
Partial	34 (79.1%)
complete	9 (20.9%)

**Table 2:** Relationship between Notch1 expression and clinicopathological data

Parameters	Notch1 expression		
	**Low (46)** **Number (%)**	**High (46)** **Number (%)**	**Test** **P-value**
Sex			
Male	24 (52.2%)	31 (67.4%)	FE *P*=0. 2
Female	22 (47.8%)	15 (32.6%)	
Age			
<55	16 (34.8%)	23 (50%)	FE *P*=0.2
>55	30 (65.2%)	23 (50%)	
Size			
<6	26(56.5%)	23 (50%)	FE *P*=0.6
>6	20 (43.5%)	23 (50%)	
Grade			
Low	35 (76.1%)	24 (52.2%)	FE *P*=0.029*
High	11 (23.9%)	22 (47.8%)	
Tumor extent			
T1	1 (2.2%)	1 (2.2%)	χ^2^ *P*=0.01*
T2	9 (19.6%)	9 (19.6%)	
T3	32 (69.6%)	19 (41.3%)	
T4	4 (8.7%)	17 (37%)	
Lymph Node			
Negative	27 (58.7%)	14 (30.4%)	FE *P*=0.011*
Positive	19 (41.3%)	32 (69.6%)	
Pathological Stage			FE *P*=0.012*
Early	28 (60.9%)	15 (32.6%)	
Advanced	18 (39.1%)	31 (67.4%)	
Vascular Invasion			FE *P*=0.014*
Negative	37 (80.4%)	25 (54.3%)	
Positive	9 (19.6%)	21 (45.7%)	
Metastasis			FE *P*= 0.001*
Negative	44 (95.7%)	30 (65.2%)	
Positive	2 (4.3%)	16 (34.8%	
Response to therapy			
Partial	9 (56.3%)	25 (96.2%)	FE *P*=0.003*
Complete	7 (43.7%)	1 (3.8%)	

FE= Fisher’s Exact

χ^2^ = Chi square

* significant

**Table 3:** Relationship between CD10 expression and clinicopathological data

Objectives	**CD10 in tumor cells**			**CD10 in stromal cells**		
	Negative (69) Number (%)	Positive (31) Number (%)	Test P-value	Low (42) Number (%)	High (37) Number (%)	Test P-value
Sex						
Male	38 (55.1%)	20 (64.5%)	FE *P*=0.5	20 (47.6%)	30 (81.1%)	FE *P*=0.002*
Female	31 (44.9%)	11 (35.5%)		22 (52.4%)	7 (18.9%)	
Age						
<55	26 (37.7%)	21 (67.7%)	FE *P*=0.09	19 (45.2%)	19 (51.4%)	FE *P*= 06
>55	43 (62.3%)	10 (32.3%)		23 (54.8%)	18 (48.6%)	
Size						
<6	39 (56.5%)	17 (54.8%)	FE *P*=0.87	27 (64.3%)	15 (40.5%)	FE *P*=0.04*
>6	30 (43.5%)	14 (45.2%)		15 (35.7%)	22 (59.5%)	
Grade						
Low	53 (76.8%)	13 (41.9%)	FE *P*=0.001*	30 (71.4%)	17 (45.9%)	FE *P*=0.024*
High	16 (23.2%)	18 (58.1%)		12 (28.6%)	20 (54.1%)	
Tumor extent						
T1	2 (2.9%)	0	χ^2^ *P*=0.002*	0	1 (2.7%)	χ^2^ *P*=0.007*
T2	20 (29%)	2 (6.5%)		9 (21.4%)	5 (13.5%)	
T3	39 (56.5%)	16 (51.6%)		29 (69%)	16(43.2%)	
T4	8 (11.6%)	13 (41.9%)		4 (9.5%)	15(40.5%)	
Lymph Node						
Negative	39 (56.5%)	6 (19.4%)	FE *P*=0.001*	20 (47.7%)	6 (16.2%)	FE *P*=0.004*
Positive	30 (43.5%)	25 (80.6%)		22 (52.4%)	31 (83.8%)	
Pathological Stage						
Early	42 (60.9%)	6 (19.4%)	FE *P*=0.001*	22(52.4%)	7 (18.9%)	FE *P*=0.002*
Advanced	27 (39.1%)	25 (80.6%)		20 (47.6%)	30 (81.8%)	
Vascular Invasion						
Negative	53 (76.8%)	17 (54.8%)	FE *P*=0.035*	33 (78.6%)	20 (54.1%)	FE *P*=0.03*
Positive	16 (23.2%)	14 (45.2%)		9 (21.4%)	17 (45.9%)	
Metastasis						
Negative	61 (88.4%)	21 (67.7%)	FE *P*=0.02	38 (90.5%)	25 (67.6%)	FE *P*=0.023
Positive	8 (11.6%)	10 (32.3%)		4 (9.5%)	12 (32.4%)	
Response to therapy						
partial	13 (65%)	21 (91.3%)	FE *P*=0.059	9(56.3%)	23 (95.8%)	FE *P*=0.004*
complete	7 (35%)	2 (8.7%)		7(43.8%)	1(4.2%)	

## Conclusion

Notch1 and CD10 expressions in colorectal carcinoma are associated with the progression of colorectal carcinoma (CRC) and partial response to treatment. They could be used as prognostic biomarkers to identify aggressive CRC. Furthermore, CD10 and Notch1 may be considered as emerging targets for CRC therapy.
